# Role of O-Acetylation in the Immunogenicity of Bacterial Polysaccharide Vaccines

**DOI:** 10.3390/molecules23061340

**Published:** 2018-06-02

**Authors:** Francesco Berti, Riccardo De Ricco, Rino Rappuoli

**Affiliations:** 1 Technical R&D, GSK Vaccines, 53100 Siena, Italy; francesco.x.berti@gsk.com; 2 Preclinical R&D, GSK Vaccines, 53100 Siena, Italy; riccardo.x.de-ricco@gsk.com; 3 External R&D, GSK Vaccines, 53100 Siena, Italy

**Keywords:** bacterial vaccines, conjugate vaccines, carbohydrate antigens, O-acetylation

## Abstract

The incidence of infectious diseases caused by several bacterial pathogens such as *Haemophilus influenzae* type b, *Streptococcus pneumoniae*, and *Neisseria meningitidis*, has been dramatically reduced over the last 25 years through the use of glycoconjugate vaccines. The structures of the bacterial capsular polysaccharide (CPS) antigens, extracted and purified from microbial cultures and obtained with very high purity, show that many of them are decorated by O-acetyl groups. While these groups are often considered important for the structural identity of the polysaccharides, they play a major role in the functional immune response to some vaccines such as meningococcal serogroup A and *Salmonella typhi* Vi, but do not seem to be important for many others, such as meningococcal serogroups C, W, Y, and type III Group B *Streptococcus*. This review discusses the O-acetylation status of CPSs and its role in the immunological responses of these antigens.

## 1. Introduction 

Glycoconjugation is a very well established ‘technology platform’ for bacterial vaccine development. Vaccines have been licensed and have strongly contributed to preventing *Haemophilus influenzae* type b, *Streptococcus pneumoniae*, and *Neisseria meningitidis* infections. Additional vaccines against other bacteria (e.g., Group B *Streptococcus*, *Salmonella typhi* Vi) or further *Streptococcus pneumoniae* and *Neisseria meningitidis* serogroups not included in the registered formulations are currently in clinical development [1]. 

Bacterial capsular polysaccharide (CPS) antigen structures, extracted and purified from microbial cultures or synthetically prepared (i.e., Quimi-Hib) [2], have been deeply investigated in the last decades. Nuclear magnetic resonance (NMR) spectroscopy, which provides fingerprint-type characteristics of the structure and is sensitive to small structural differences, has played a key role in the carbohydrate structure determination [3].

Bacterial polysaccharides often contain an array of substituents, such as O-acetyl and phosphate groups, which may constitute an important part of the immunodominant epitopes. CPSs from many pathogenic bacteria are O-acetylated, and a common feature is that the O-acetyl groups are easily removed at an alkaline pH. The O-acetyl content is considered an aspect of ‘identity’, as some immunologically distinguishable CPSs differ in the presence of O-acetyl groups only (e.g., pneumococcal types 9A–9V [4], and 11A–11E [5,6]); the immune responses evoked by these CPSs are not necessarily cross-protective.

**Figure 1:**
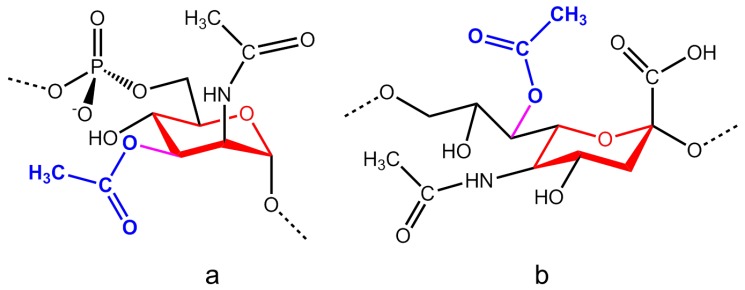
Repeating unit structures of meningococcal serogroup (**a**) A and (**b**) C, with O-acetyl groups inside or outside the saccharide ring, respectively.

O-acetyl groups have been determined to be inside or outside the saccharide ring of monosaccharides constituting the repeating units of capsular polysaccharides. In Figure 1, the repeating unit structures of meningococcal serogroup A and C with O-acetyl groups inside or outside the saccharide ring are respectively reported.

Here we review the presence of the O-acetyl groups in capsular antigens and we try to address their role in the functional immune response.

## 2. O-acetylation in Conjugate Vaccines

O-acetylation patterns have been determined for several polysaccharide antigens (Table 1). 

### 2.1. Neisseria Meningitidis

The α-1,6-linked *N*-acetylmannosamine phosphate constituting the repeating unit of meningococcal serogroup A CPS is partly O-acetylated at position C_3_ or C_4_ (Table 1)_._ The meningococcal serogroups C, W, and Y, which all contain Neu5Ac moiety in the relative repeating units, are partially O-acetylated at the glycerol chain of Neu5Ac (at positions C_7_ and C_8_ for serogroup C and at positions C_7_ and C_9_ for serogroup W and Y) [7] (Table 1). The meningococcal genes coding O-acetylation of various serogroups have been analyzed [8]. On the contrary, the meningococcal serogroup X CPS [→4)-α-d-Glc*p*NAc-(1→OPO_3_→] is not O-acetylated [9,10]. 

The meningococcal serogroup A plain CPS is largely O-acetylated at position C_3_ (>90%) and slightly at position C_4_ [11,12] (Figure 1a). MynC was identified as new subclass of O-acetyltransferases that utilize acetyl-CoA to decorate the serogroup A CPS. Although a single or dual specificity of MynC (at positions C_3_ and/or C_4_) has not been demonstrated, the single specificity and a spontaneous migration of the O-acetyl group from the site of attachment around the ring is, perhaps, a more likely explanation. While a study demonstrated that O-acetylation is not required for capsule expression or to protect the meningococci from killing by normal human sera, investigation with O-acetylation-deficient mutant confirmed the importance of O-acetylation in serogroup A polysaccharide immunogenicity [11,13,14]. Berry et al. [15] confirmed that serogroup A-specific antibodies elicited in post-immunization human sera mostly identified O-acetylated CPS residues, impacting the degree of antibody inhibition. Comparative immunogenicity studies in mice revealed that de-O-acetylated antigens resulted in a marked loss of immunogenicity (total IgG), and, most relevantly, a loss in their ability to induce functional bactericidal antibodies. However, epitopes not involving O-acetyl groups may also contribute to the development of protective response since mice vaccinated with de-O-acetylated antigens elicited some functional antibodies.

In a phase III partially blinded, controlled study (where 1170 healthy subjects aged 18 to 25 years were randomized (1:1:1)), two lots of meningococcal ACWY-tetanus toxoid conjugate vaccines (MenACWY-TT) that differed in serogroup A CPS O-acetylation levels respectively (68% vs. 92% O-acetylation), were evaluated in terms of immunogenicity (i.e., rabbit serum bactericidal activity (rSBA)) and safety in comparison to a licensed MenACWY CPS vaccine (82% serogroup A O-acetylation). The MenA-TT conjugate with O-acetylation levels of 68% and 92% resulted in comparable vaccine immunogenicity [16]. As a matter of fact, all the meningococcal serogroup A conjugate contained in licensed vaccines (MenAfriVac–Serum Indian Institute; Menveo–GSK Vaccines; Menactra–Sanofi Pasteur; Nimenrix–GSK Vaccines (later taken over by Pfizer)) are largely O-acetylated. However, no evidence has been collected to define the minimal O-acetylation level affecting the immunogenicity of meningococcal serogroup A vaccines for the moment.

The high relevance of the O-acetyl moieties for serogroup A *N. meningitidis* could also be related to the presence of this decorative group within the saccharide ring, probably influencing and specifying the epitope in a unique arrangement that could not be easily mimicked in its absence.

Alternatively, the meningococcal serogroup C CPS is highly O-acetylated (>90%) at positions C_8_ and C_7_ of the sialic acid glycerol chain (out of the ring) (Figure 1b). The *oatC* gene required for O-acetylation of sialic acid residue was identified [8]. In meningococcal C CPSs freshly extracted or not exposed to chemical conditions (i.e., basic pH), which facilitates the migration from/to vicinal hydroxyl groups (i.e., at C_8_ and C_7_), most of the O-acetylation exists at position C_8_ with fewer CPSs repeating O-acetylation at C_7_, or results de-O-acetylated. After migration, most of the O-acetyl groups relocate from C_8_ to C_7_, leading to an epitope that is conformationally related, but not identical, to one contained in the fully de-O-acetylated serogroup C CPS. As demonstrated by Michon et al. [17] the immunogenicity in mice vaccinated with partially or completely de-O-acetylated serogroup C CPS antigens, obtained by mild basic treatment of the antigens, results in higher SBA titers toward the O-acetylated serogroup C strain C11. This strain was the dominant one for the de-O-acetylated antigen during the late 1990s in the UK. However, clinical data confirmed that both O-acetylated (Menjugate, Menveo, Menitorix, Menhibrix–GSK Vaccines; Menactra–Sanofi Pasteur; Nimenrix–Pfizer) and de-O-acetylated (NeisVac–North American Vaccine Inc., later taken over by Baxter Bioscience) conjugates were very immunogenic and efficacious in large vaccination campaigns [18,19,20,21] independent of the used carrier protein (TT, DT, CRM_197_). The high immunogenicity did not directly correlate to the C_7_ and C_8_ O-acetylation in meningococcal serogroup C. This differs from the serogroup A in which the O-acetylation seems to be determinant in the immunoresponse. This lack of correlation might be related to the positions of the O-acetyl groups which are located in the glycerol chain outside the saccharide ring where the influence on the epitope could be lower. 

For meningococcal W and Y CPSs, minimal O-acetylation levels have been also revealed at positions C_7_ and C_9_ of sialic acid moieties of several strains. As suggested by Fusco et al. [22], for the meningococcal Y CPSs the O-acetyl is likely present on the surface of the organism and it seems to display significant conformational differences around its sialic acid linkages. This is not the same for the glycosidic bond between C_6_ glucose and C_2_ sialic acid, which does not seem to be affected by the O-acetylation status of the sialic acid. Furthermore, the authors proposed that conformational differences around the sialic acid bond between the C_7_ and C_9_ O-acetylated forms could be explained by the fact that the O-acetyl located at C_7_, in addition to the linkage at C_4_, influences the rotation of the sialic acid glycosidic bonds. One the other hand, the O-acetyl at C_9_, being away from these two centers, has no influence on the rotation (torsional angle), which therefore allows the conformational identity between C_9_ O-acetylated and de-O-acetylated forms of Y CPSs. The preferred position of the O-acetyl group on C_9_ Neu5Ac, which has been detected as most abundant form after purification of both meningococcal W and Y CPSs [12], might arise from its higher thermodynamic stability and as a consequence of the migration of O-acetyl group from the C_7_ to C_9_ positions. 

Although the natural occurrence of both O-acetylated and non-O-acetylated W and Y isolates has been demonstrated (i.e., in the UK with a predominance of non-O-acetylated W and O-acetylated Y strains [23], and during the Hajj outbreak where all the W strains were non-O-acetylated [24]), the currently licensed serogroup W and Y conjugate vaccines contain the O-acetylated CPS. The general role of O-acetylation of these CPSs is still debated, however several studies conducted in the preclinical stage and using human sera seem to confirm that there is no difference in functional activity of O-acetylated or non-O-acetylated CPSs [22,25,26,27]. By investigating the meningococcal Y antigen, Fusco et al. [22] suggested that the O-acetyl groups may mask an important epitope to the immune system and thereby mislead the antibody response, resulting in an escape mechanism. Similar to meningococcal serogroup C, the O-acetylation in serogroup W and Y is outside the saccharide ring. It might confirm the poor immunological impact of this group when positioned outside the saccharide ring. However, the relatively low level of O-acetylation of several meningococcal serogroup W and Y strain might also explain the poor impact.

### 2.2. *Group B* Streptococcus

O-acetylation patterns at the ‘glycerol chain’ of the Neu5Ac residue, present at the terminal end of the branch, were also confirmed for several Group B *Streptococcus* (GBS) CPS serotypes (Ia, Ib, II, III, V, and VI) (Table 1), and were demonstrated to affect the inhibition of neutrophil suppression and virulence [28,29,30]. Data were consistent with an initial O-acetylation at position C_7_, and subsequent migration of the O-acetyl ester at positions C_8_ and C_9_. As for mammalian glycoproteins, O-acetylation of terminal sialic acid is a mechanism to prevent enzymatic de-sialylation; the exposure of terminal galactose and its excretion from the animal allow for speculation on a similar attitude of GBS.

Sera from healthy adults immunized with de-O-acetylated GBS CPS-TT conjugate vaccines were evaluated in opsonophagocytosis assays using 20 GBS clinical isolates (type Ia, Ib, II, III, or V CPSs with O-acetylation levels ranging from 2 to 40%). The data showed >90% opsonophagocytosis and killing of all strains, confirming that de-O-acetylated CPS-conjugate vaccines contain immunogenic epitopes that offer protection against GBS, independent of O-acetyl CPS modifications. Thus, presence of O-acetyl groups on the GBS CPSs is not essential for functional antibodies to be elicited by GBS glycoconjugate vaccines [31]. In the work of Carboni et al. the structure of a protective epitope of the group B *Streptococcus* type III capsular polysaccharide was investigated. The authors observed that the mAb interacts with the O_7_ of sialic acid through an H_2_O molecule, and dimensionally, this space could also host an acetyl group [32]. This could explain data in the literature reporting that O-acetylation does not interfere with protection. 

### 2.3. Streptococcus Pneumoniae

The presence of O-acetyl groups has been observed in several *S. pneumoniae* CPSs (Table 1) but in only some has the potential role of O-acetylation been explored. A comparative study conducted both in the infant rhesus monkey model and in humans confirmed that antibodies against the de-O-acetylated backbone as well as against the O-acetylated serotype 9V CPS (O-acetyl groups revealed at positions C_2_ and C_3_ of the Glc residue and at positions C_4_ and C_6_ of the Man*p*NAc residue) [33,34] were detected. On the other hand, opsonophagocytic functional activity was observed in antisera in which the predominant antibody species recognize the de-O-acetylated serotype 9V CPS. The authors concluded that the O-acetylation side groups, while being recognized, were not essential to the ability of the serotype 9V CPS to elicit a functional antibody response [34].

For the serotype 18C CPS, which contains O-acetyl groups at position C_6_ of Glc residue in the branch [35], the role of O-acetylation was explored by using rabbit and human sera. No differences were shown in inhibition curves between any of the analyzed sera and CPSs with different degrees of O-acetylation [36].

### 2.4. Salmonella Typhi *Vi*

The α-1,4-linked *N*-acetylgalacturonic acid constituting the repeating unit of *Salmonella typhi* Vi CPS is partly O-acetylated (60–90%) at position C_3_ (Table 1)_._ The O-acetylation level is one of the critical determinants of immunogenicity in the Vi polysaccharide antigen. Removal of O-acetyl groups resulted in a lower vaccine immunogenicity [37,38]. 

The molecular model of a pentamer (five repeating units) showed that the bulky nonpolar O-acetyl groups at position C_3_ make up most of the surface of Vi CPS by protruding on both sides. The O-acetyl groups dominate the surface of Vi CPS and it might explain their relevant influence on its immunogenicity [39].

### 2.5. Staphylococcus Aureus

Both *S. aureus* type 5 and 8 CPSs contain Man*p*NAcA and L- and D-Fuc*p*NAc residues (Table 1). The two CPSs differ in the stereochemical glycosidic linkages between the monosaccharides constituting the repeating units and the site of O-acetylation. The sites of O-acetylation are at the position C_3_ of L-Fuc*p*NAc for type 5 and at the position C_4_ of d-Man*p*NAcA type 8 [40]. 

As confirmed by Scully et al. [41] by collecting in vivo (mouse model) and in vitro experiments, the O-acetylation is necessary for CPS-CRM_197_ conjugates to induce effective opsonophagocytic killing responses. This data supports the high importance of a careful monitoring of the degree of O-acetylation during vaccine development and production. 

The authors suggested that this finding may provide additional insight into the previous failure of a *S. aureus* CPS conjugate vaccine. Clashing with a previous clinical trial conducted in a similar population, which suggested short-term efficacy (up to 40 weeks) against *S. aureus* bacteremia after a single dose of a bivalent vaccine [42] (StaphVAX–Nabi; CPS types 5 and 8 conjugated with *Pseudomonas aeruginosa* exotoxin A) [43], a larger Phase III study failed despite robust antibody responses to primary vaccination. There was no demonstrative vaccine efficacy in preventing *S. aureus* bacteremia in end-stage renal disease patients receiving hemodialysis at any time interval studied or in any strategy considered [44]. There has been speculation on the reasons behind this failure [41,44]. Issues in the manufacturing consistency of the Phase III clinical trial material have been considered due to changes in contract facilities used, although the specific impacts of these changes were not further explained. Access to the material used in the phase 3 study, for instance to detect the potentially important critical quality attributes such as O-acetylation status, would be useful for a direct comparison with earlier results and to further correlate those data with recent evidence collected in the preclinical model [42]. 

## 3. Conclusions and Future Perspectives

The O-acetyl content of CPSs so far has not been considered an attribute to be monitored for quality control in the manufacturing of glycoconjugate vaccines. Therefore, content in vaccines may vary for several reasons, including the potential release of O-acetyl groups over time in solution because the group is labile in certain conditions (preferentially in a basic environment or as a consequence of repeated freeze and thawing). Also, variety in the initial content which may be attributed to different growth conditions and manufacturing processes (i.e., different manufacturers and lot-to-lot consistency).

By summarizing the available literature data around O-acetylation, a common driver could be speculated upon: for those antigens comprised of a repetition of single or few saccharide repeating units (e.g., meningococcal serogroup A, *Staphylococcus* types 5 and 8), the position and the role of the O-acetyl group weighs more on the overall structure due to the fact that the epitope might be involved in the repetition of the same saccharide. On the other hand, as confirmed by data available on meningococcal serogroup C, Y or Group B *Streptococcus* type III antigens, where the O-acetyl group is located on a ’glycerol’ chain out of the ring positions, its presence seems to be less relevant to the immunological response. This could also explain why a migration to vicinal sites of the O-acetyl group (e.g., in C_7_ from C_8_ of meningococcal serogroup C CPS) does not influence the immunoresponse.

Although the position of O-acetyl group is inside or outside the saccharide ring, its distribution and population in terms of percentage of O-acetylated portions should always be considered as a critical quality attribute for a candidate vaccine.

In several cases, including meningococcal serogroup A and pneunomococcal serotype 1, the presence of the O-acetyl groups prevents periodate oxidation and reductive amination coupling as a manufacturing strategy for the glycoconjugates. Controlled de-O-acetylation is required to produce vaccines by this route (e.g., MenAfriVac, Serum Institute India, PATH). For this reason, the O-acetylation pattern also may influence the choice of manufacturing strategy.

The O-acetyl content of CPSs has been historically determined by the Hestrin colorimetric assay [45]. However, during the last 15–20 years new advanced methods that are sufficiently sensitive and based on nuclear magnetic resonance (NMR) spectroscopy [12,46,47] or anion exchange chromatography coupled with conductivity detection (HPAEC-CD) [48] have been developed. While colorimetric assay and HPAEC-CD provides an overall estimation of O-acetyl content, NMR assay developed for meningococcal CPSs [12] also provides a precise distribution of the O-acetyl content at the different saccharide positions. For antigens eliciting an immune response dependent upon the O-acetyl status, it is considered as a key stability attribute which should be monitored carefully. 

As reported by Jones et al. [46] and Lemercinier et al. [47], the kinetics of O-acetyl group release following a basic treatment with 0.2 M NaOD, which might be an indicator of its stability in aqueous solution, resulted more rapid (typically 20 min after NaOD addition) for meningococcal serogroups A, W, and Y, while it appeared slower for meningococcal serogroup C (the sample was typically incubated at 37°C for 1 h for complete de-O-acetylation) and for *Salmonella typhi* Vi CPSs, and also showed that with a 0.05 M final base concentration, the progress of de-O-acetylation could be followed in the spectrometer (narrowing of the CPS NMR peaks). 

In conclusion, today we have tools to perform precise quantitative measurements of O-acetyl content in CPSs, and we should consider it a fundamental attribute to be monitored for quality control. However, each bacterial polysaccharide antigen should be considered and analyzed independently due to the intrinsic behavior that each one can exhibit. As shown for the selected bacterial polysaccharide antigens (*Neisseria meningitidis*, Group B *Streptococcus*, *Streptococcus pneumoniae*, *Salmonella typhi* Vi *and Staphylococcus aureus*) that have been used in licensed vaccines or vaccine clinical trials, data in the literature strongly demonstrate the fundamental role of the O-acetyl moieties influencing the immune response and, in some cases, imposing a different conformational epitope.

In the future, the ability to determine the crystal structure of protective epitopes [32] combined with the possibility of preparing synthetic conjugate vaccines and the precision of analytical tools such as NMR will allow for a better understanding of the role of O-acetyl groups in glycoconjugate vaccines.

## Figures and Tables

**Table 1:** Structures of the repeating units containing O-acetyl groups of bacterial polysaccharides antigens of vaccines licensed or tested in clinical trials.

Polysaccharide	Repeat Unit
*Neisseria meningitidis*	
Group A	→6)-α-d-Man*p*NAc(3/4OAc)-(1→OPO_3_→
Group C	→9)-α-d-Neu5Ac(7/8OAc)-(2→
Group W	→6)-α-d-Gal*p*-(1→4)-α-d-Neu5Ac(7/9OAc)-(2→
Group Y	→6)-α-d-Glc*p*-(1→4)-α-d-Neu5Ac(7/9OAc)-(2→
Group B *Streptococcus*	
Type Ia	→4)-[α-d-Neu5Ac(7/8/9OAc)-(2→3)-β-d-Gal*p*-(1→4)-β-d-Glc*p*NAc-(1→3)]-β-d-Galp-(1→4)-β-d-Glc*p*-(1→
Type Ib	→4)-[α-d-Neu5Ac(7/8/9OAc)-(2→3)-β-d-Gal*p*-(1→3)-β-d-Glc*p*NAc-(1→3)]-β-d-Gal*p*-(1→4)-β-d-Glc*p*-(1→
Type II	→3)-β-d-Glc*p*-(1→2)-[α-d-Neu5Ac(7/8/9OAc)-(2→3)]-β-d-Gal*p*-(1→4)-β-D-Glc*p*NAc-(1→3)-[β-d-Gal*p*-(1→6)]-β-d-Gal*p*-(1→4)-β-d-Glc*p*-(1→
Type III	→6)-[α-d-Neu5Ac(7/8/9OAc)-(2→3)-β-d-Gal*p*-(1→4)]-β-d-Glc*p*NAc-(1→3)]-β-d-Gal*p*-(1→4)-β-d-Glc*p*-(1→
Type V	→4)-[α-d-Neu5Ac(7/8/9OAc)-(2→3)-β-d-Gal*p*-(1→4)-β-d-Glc*p*NAc-(1→6)]-α-d-Glc*p*-(1→4)-[β-d-Glc*p*-(1→3)]-β-d-Gal*p*-(1→4)-β-d-Glc*p*-(1→
Type VI	→6)-[α-d-Neu5Ac(7/8/9OAc)-(2→3)-β-d-Gal*p*-(1→3)]-β-d-Glc*p*-(1→3)-β-d-Gal*p*-(1→4)-β-d-Glc*p*-(1→
*Streptococcus pneumoniae*	
Type 1	→3)-d-AAT-α-Gal*p*-(1→4)-α-d-Gal*p*A(2/3OAc)-(1→3)-α-d-Gal*p*A-(1→
Type 7F	→6)-[β-d-Gal*p*-(1→2)]-α-d-Gal*p*-(1→3)-β-L-Rha*p*(2OAc)-(1→4)-β-d-Glc*p*-(1→3)-[α-d-Glc*p*NAc-(1→2)-α-L-Rha*p*-(1→4)]-β-d-Gal*p*NAc-(1→
Type 9V	→4)-α-d-Glc*p*(2/3OAc)-(1→4)-α-d-Glc*p*A-(1→3)-α-d-Gal*p*-(1→3)-β-d-Man*p*NAc(4/6OAc)-(1→4)-β-d-Glc*p*-(1→
Type 15B	→6)-[α-d-Gal*p*(2/3/4/6OAc)-(1→2)-[Gro-(2→P→3)]-β-d-Gal*p*-(1→2)]-β-d-Glc*p*NAc-(1→3)-β-d-Gal*p*-(1→4)-β-d-Glc*p*-(1→
Type 17F	→3)-β-L-Rha*p*-(1→4)-β-d-Glc*p*-(1→3)-α-d-Gal*p*-(1→3)-β-L-Rha*p*(2OAc)-(1→4)-α-L-Rha*p*-(1→2)-d-Ara-ol-(1→P→
Type 18C	→4)-β-d-Glc*p*-(1→4)-[α-d-Glc*p*(6OAc)-(1→2)][Gro-(1→P→3)]-β-d-Gal*p*-(1→4)-α-d-Glc*p*-(1→3)-β-L-Rha*p*-(1→
Type 22F	→4)-β-d-Glc*p*A-(1→4)-[α-d-Glc*p*-(1→3)]-β-L-Rha*p*(2OAc)-(1→4)-α-d-Glc*p*-(1→3)-α-d-Gal*f*-(1→2)-α-L-Rha*p*-(1→
Type 33F	→3)-β-d-Gal*p*-(1→3)-[α-d-Gal*p*-(1→2)]-α-d-Gal*p*-(1→3)-β-d-Gal*f*-(1→3)-β-d-Glc*p*-(1→5)-β-d-Gal*f*(2OAc)-(1→
*Salmonella enterica*	
*typhi* Vi	→)-α-d-Gal*p*NAcA(3OAc)-(1→
*Staphylococcus aureus*	
Type 5	→4)-β-d-Man*p*NAcA-(1→4)-α-L-Fuc*p*NAc(3OAc)-(1→3)-β-d-Fuc*p*NAc-(1→
Type 8	→3)-β-d-Man*p*NAcA(4OAc)-(1→3)-α-L-Fuc*p*NAc-(1→3)-α-d-Fuc*p*NAc-(1→

Abbreviations: Glc, Glucose; Gal, Galactose; Neu5Ac, *N*-acetyl neuraminic acid (sialic acid); Rha, Rhamnose; GlcNAc, *N*-acetyl Glucosamine; GalNAc, *N*-acetyl Galactosamine; FucNAc, *N*-acetyl Fucosamine; ManNAcA, *N*-acetyl Mannuronic Acid; AAT, 2-acetamido-4-amino-2,4,6-trideoxygalactose; Gro, glycerol, Pne, 2-acetamido-2,6-dideoxytalose; Sug, 2-acetamido-2,6-deoxyhexose-4-ulose; P, phosphate in a phosphodiester linkage.
